# Seed Dispersal and Establishment of Endangered Plants on Oceanic Islands: The Janzen-Connell Model, and the Use of Ecological Analogues

**DOI:** 10.1371/journal.pone.0002111

**Published:** 2008-05-07

**Authors:** Dennis M. Hansen, Christopher N. Kaiser, Christine B. Müller

**Affiliations:** Institute of Environmental Sciences, University of Zurich, Zurich, Switzerland; Centre National de la Recherche Scientifique, France

## Abstract

**Background:**

The Janzen-Connell model states that plant-specific natural enemies may have a disproportionately large negative effect on progeny close to maternal trees. The majority of experimental and theoretical studies addressing the Janzen-Connell model have explored how it can explain existing patterns of species diversity in tropical mainland areas. Very few studies have investigated how the model's predictions apply to isolated oceanic islands, or to the conservation management of endangered plants. Here, we provide the first experimental investigation of the predictions of the Janzen-Connell model on an oceanic island, in a conservation context. In addition, we experimentally evaluate the use of ecological analogue animals to resurrect the functional component of extinct frugivores that could have dispersed seeds away from maternal trees.

**Methodology/Principal Findings:**

In Mauritius, we investigated seed germination and seedling survival patterns of the critically endangered endemic plant *Syzygium mamillatum* (Myrtaceae) in relation to proximity to maternal trees. We found strong negative effects of proximity to maternal trees on growth and survival of seedlings. We successfully used giant Aldabran tortoises as ecological analogues for extinct Mauritian frugivores. Effects of gut-passage were negative at the seed germination stage, but seedlings from gut-passed seeds grew taller, had more leaves, and suffered less damage from natural enemies than any of the other seedlings.

**Conclusions/Significance:**

We provide the first experimental evidence of a distance-dependent Janzen-Connell effect on an oceanic island. Our results potentially have serious implications for the conservation management of rare plant species on oceanic islands, which harbour a disproportionately large fraction of the world's endemic and endangered plants. Furthermore, in contrast to recent controversy about the use of non-indigenous extant megafauna for re-wilding projects in North America and elsewhere, we argue that Mauritius and other oceanic islands are ideal study systems in which to empirically explore the use of ecological analogue species in restoration ecology.

## Introduction

Animal-mediated seed dispersal and subsequent differences in seedling establishment and survival in relation to distance from adult conspecific plants are important factors in the dynamics of tropical forests [e.g. 1]. This has been intensely studied during the last three decades in the framework of the Janzen-Connell model [2]–[4], which states that host-specific seed predators, or seedling herbivores and pathogens may have a disproportionately large negative effect on progeny close to maternal trees. However, the vast majority of both experimental and theoretical studies focusing on the Janzen-Connell model have been primarily concerned with exploring the origin and maintenance of patterns of species diversity, and were less concerned with the potential importance of this pattern for conservation ecology [reviewed in e.g. 5].

In our study we experimentally address the Janzen-Connell model in a conservation context on tropical islands, where endangered plants are often found in very low numbers within small areas. Furthermore, we assess the use of ecological analogue seed-dispersing animal species to resurrect the functional component of extinct endemic frugivores.

### The Janzen-Connell model on oceanic islands

Two key points emerge in relation to the Janzen-Connell model and how it could apply to the ecology and conservation of plants in oceanic island ecosystems. Firstly, patterns of seed- and seedling mortality on islands may be different from those found in mainland ecosystems. Generally, a high host-specificity of herbivores and pathogens is an assumption of the Janzen-Connell model of seedlings and sapling distribution [4]. Island ecosystems are often simpler than mainland ecosystems, in which case we would expect more generalist than specialist seed predators and herbivores on islands than on the mainland. If so, we could expect Janzen-Connell patterns to be less prevalent on oceanic islands than in comparable mainland habitats. There have been many studies on specialist versus generalist herbivorous insects in tropical forests [reviewed in 6], but very little is known about the relative importance of specialist and generalist insect herbivores on oceanic islands [7]. The second key point is that studies of Janzen-Connell patterns are more urgent in relation to conservation management for oceanic islands than for most mainland ecosystems. Pristine oceanic islands typically harboured fewer species of frugivorous vertebrate than comparable mainland areas, and many of those few species are now extinct [8]. Thus, on most oceanic islands the frugivorous members of the afterlife [*sensu* 9] currently outnumber the living, and many seed dispersal interactions are likely to have been lost. Today, the remaining native and endemic flora and fauna of most oceanic islands are often crammed into much smaller remnant patches of native habitats than those on the mainland. Therefore, if Janzen-Connell patterns are indeed prominent on oceanic islands, endangered plant species relying on vertebrate dispersal by now-extinct animals face a double peril: not only do they lack most of the agents that once mediated the escape and establishment of their progeny away from maternal trees, but present-day native habitats may be too small to support viable populations of plants that exhibit strong Janzen-Connell spacing patterns as a result of actions of natural enemies.

Only few studies have investigated aspects of seed dispersal and seed- and seedling survival in the framework of the Janzen-Connell model on oceanic islands (Galápagos [10], other Pacific islands [11], 12, and the Canary Islands [13]). Most of them showed that saplings and juvenile trees are found away from adult trees; however, in the Canary Islands Arevalo and Fernandez-Palacios [13] found no effect of distance to conspecific adults on sapling density, suggesting that Janzen-Connell spacing plays a minor role in this forest. None of these studies were experimental, however, but analysed patterns in natural plant populations. Despite a long scientific history of using islands as natural laboratories for ecological and evolutionary studies [e.g. 14], [15], we are not aware of any studies that have experimentally investigated seed germination and the post-germination fate of seedlings in the framework of the Janzen-Connell model on oceanic islands. Consequently, while it is acknowledged that oceanic islands harbour a disproportionally large fraction of the most critically endangered plant species in the world, we know next to nothing about how one of the most widely studied ecological patterns affects the regeneration and longer-term survival of these plants.

### Ecological analogue species and the resurrection of extinct interactions

One way of recreating lost seed dispersal dynamics is to introduce extant proxy species—ecological analogues or taxon substitutes—that perform similar ecosystem functions as extinct ones. To some ecologists and conservation biologists this idea may be anathema; in a best-case scenario it could be seen as little more than an attempt to create a small-scale version of Jurassic Park, and in one worst-case scenario it runs the risk of introducing species that may become invasive and have unintended negative effects on the ecosystem. Recently, the use of ecological proxies to recreate the Pleistocene megafauna in South and North America, and in the Sibirian Tundra has been the subject of a heated debate [16]–[22], partly due to the complexity of the involved ecosystems, and partly because of the vast areas needed to sustain populations of the suggested large-bodied animals. In contrast, due to their relatively small size and relative simplicity of their native ecosystems, oceanic islands are ideal systems in which to empirically explore the use of ecological analogue species in a conservation management context [23]–[25].

### Our study

Our model system was the oceanic island of Mauritius, which faces most of the problems that affect oceanic island ecosystems. Mauritius has lost the majority of its original vertebrate frugivorous and seed-dispersing fauna, and some studies have suggested that missing seed dispersers could be contributing to the continued decline in many of the endangered Mauritian plant species today [26], [27]. Nevertheless, the role of extant or extinct seed dispersal interactions in forest dynamics in present-day Mauritius has not been directly addressed so far [but see 28]. We used the critically endangered endemic tree *Syzygium mamillatum* (Myrtaceae) as our model organism to study the effect of missing seed dispersers in the dynamics of present-day native forests in Mauritius.

We focused on seed germination, and the establishment and survival of seedlings of *S. mamillatum*. We addressed the specific questions: Are seed germination, and seedling growth and survival of *S. mamillatum* affected by distance to maternal trees? If so, can we use extant frugivorous animals as ecological analogue species to resurrect lost forest dynamics and ameliorate these negative effects?

## Materials and Methods

### Study site

The study was conducted in the Black River Gorges National Park in Mauritius between March 2004 and February 2006, in a 24 ha fenced and weeded Conservation Management Area (CMA) that was established in the lower montane evergreen wet forest of Brise Fér in 1996 (20°22.5′S, 57°26′E, 570–600 m elevation). Outside the CMA, the native forest is heavily degraded by invasive species. The CMA is roughly divided by a steep slope of 15–25 m in height into an upper southwestern plateau, characterised by a thin layer of top soil (erosion area), and a lower northern and northeastern plateau with deeper soil (accumulation area). Reflecting this, on the upper plateau the forest is relatively low (8–12 m), while it is taller on the lower plateau (15–25 m). The forest on the upper plateau is drier and more open than the forest on the lower plateau.

### Study species

*Syzygium mamillatum* is a critically endangered endemic upland sub-canopy tree of 2.5–9 m in height. Despite the striking basal cauliflory of *S. mamillatum*, the species was not described until 1987 [29]. During a focused search in July and August 2003 we found a total of 119 mature trees in Brise Fér – representing the largest known population with 87% of all known mature trees of this species, with an additional few single trees or small stands elsewhere in the National Park. The majority of trees in Brise Fér occur within the CMA (81 trees = 68%). Of the 81 adult trees in the CMA, 79 were upright and the remaining two trees had been partly knocked over by falling trees. Of the upright trees, 58 grew on the lower plateau and 21 on the upper plateau. The difference between upper and lower plateau in the CMA is apparent in the size of adult *S. mamillatum* trees. Trees growing on the lower plateau are larger than trees growing on the upper plateau, in terms of both height (all values are mean±SD, compared with Student's t-tests; lower plateau = 6.2±1.4 m, upper plateau = 5.2±1.3 m, t = 2.911, *P* = 0.006) and diameter at breast height (lower plateau = 6.8±2.2 cm, upper plateau = 5.3±1.7 cm, t = 3.08, *P* = 0.004).

Despite intensive searches throughout the study period, no natural seedlings or saplings of *S. mamillatum* were found further than 1–2 m away from adult trees. We found the tallest natural seedlings (30–40 cm) around the three adult trees in the ‘Old Plot’ – a 1-ha plot in the Brise Fér CMA, which has been weeded since 1987. However, these were all in a bad shape with only a handful of heavily damaged leaves left.

**Figure 1:**
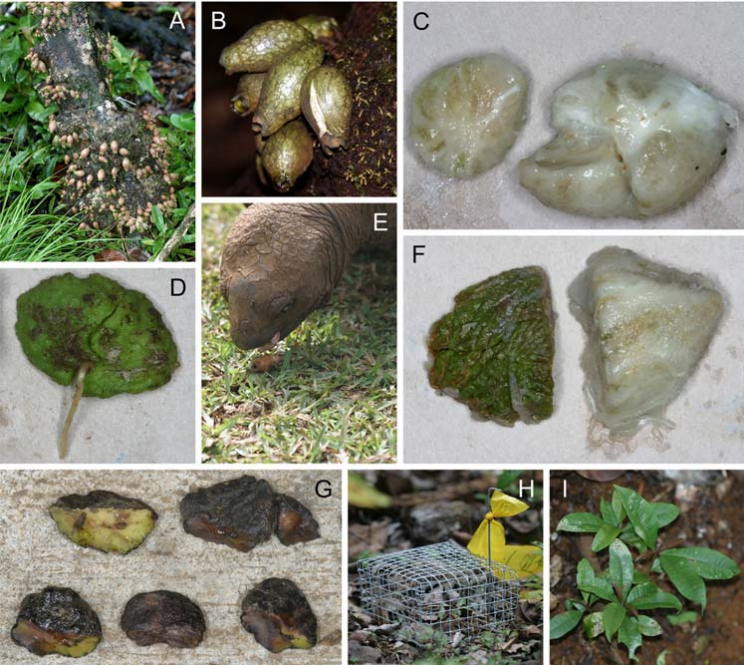
Fruits, seeds, and seedlings of *Syzygium mamillatum*. (A) Developing fruits on the lower ∼50 cm of a *Syzygium mamillatum* tree. (B) Ripe fruits attached to the trunk. Note the foremost fruit has split open, releasing a fermented smell. (C) A ‘ball’ of four seeds from one fruit with the pulp removed. (D) Germinating seed. Note the clear line between the two green cotyledons. (E) Giant Aldabra tortoise feeding on *S. mamillatum* fruits. (F) Seeds with and without the slimy, fibrous endocarp. (G) Seed fragments after tortoise gut-passage. Fragments were most often found as whole cotyledons. Note how some cotyledons are still green on the side that faced the other cotyledon, suggesting that they did not break apart until late in the passage. (H) A caged patch of seeds. (I) An experimental patch of seedlings.

In another study [30], we investigated the pollination biology of *S. mamillatum*, and found it to be pollinated by endemic and introduced bird species. The average fruitset of trees in the CMA was 20–25%, with trees producing 1–520 ripe fruits (mean±SD: 48±100 fruits; N = 69 trees). Interestingly, on average, 73% of all ripe fruits on a tree developed on the lowest 30 cm of the trunk (Figure 1A). For further details on fruit- and seed structure, please refer to Figure 1. No seedlings of native or endemic plant species survive to sapling stage in the heavily invaded forest outside the CMAs, due to both competition with invasive plants and grazing by introduced animals [31, Mauritian Wildlife Foundation unpublished database]. Therefore, it is of greatest applied conservation importance to investigate and attempt to re-establish some of the lost dynamics within the CMAs.

### Statistical analyses

Statistical models and methods used are specified in the relevant sections. All analyses were done with R.2.4.1 [32].

### Feeding experiments with ecological analogue species

Out of the multitude of frugivorous seed-dispersing ghosts in the Mauritian fauna [33], [27], we selected to resurrect and investigate the functional component of three of them, the dodo (*Raphus cucullatus*) and the two species of giant tortoises, the high- or saddle-backed tortoise (*Cylindraspis triserrata*) and the domed tortoise (*C. inepta*). As dodo stand-ins, we used three domestic turkeys (*Meleagris gallopavo*). Turkeys have a powerful gizzard with grinding stones, like the dodo had [34], and no *S. mamillatum* seeds passed through unharmed (N = 105 fruits); we only found seed fragments of 1–2 mm in size. Therefore, we conclude that turkeys are not suitable analogue seed dispersers for *S. mamillatum*, and we present no further data from this part. As stand-in for the two extinct giant tortoise species of Mauritius we used giant Aldabra tortoises, *Aldabrachelys gigantea*. All extinct Mascarene giant tortoise species have been reported to eat fruits and leaves [reviewed in 27]. Similarly, the Aldabra tortoise feeds on different plant material, including fruits, and acts as a seed disperser for several plant species in Aldabra [35].

For the feeding experiment, we used three giant Aldabra tortoises from La Vanille Crocodile and Tortoise Park, Riviére des Anguilles (La Vanille hereafter). The medium-sized tortoises, weighing approximately 70–100 kg each, were kept together in an enclosure, where they were also being fed vegetables and other fruit throughout the feeding experiment. Forty *S. mamillatum* fruits were fed to the three tortoises twice a week during four weeks, beginning on March 10, and finishing on April 5. A total of 320 ripe fruits from seven different *S. mamillatum* trees were fed to the tortoises (mean = 46 fruits/tree, range: 20–132 fruits/tree). *Syzygium mamillatum* fruits were fed whole to the tortoises (Figure 1E). We estimated that the fruits fed to the tortoises contained a total of 685 seeds based on the average number of 2.14 seeds per fruit [30]. Tortoise faeces were collected daily in plastic bags at La Vanille from March 11 to May 5. Once a week, we collected the bags from La Vanille and examined the faeces. Whole *S. mamillatum* seeds and seed fragments, which were large enough to be identified as such (Figure 1G), were extracted, counted and weighed.

### Germination experiments

We set up two different seed germination experiments in Brise Fér CMA. One in which we used whole fruits and manually depulped seeds, and another where we used tortoise gut-passed seeds from the feeding experiment.

For the first experiment, with whole fruits and manually depulped seeds, an unbalanced factorial design with four treatments was set up around 20 fruit-bearing *S. mamillatum* trees (if not stated otherwise, the replication for lower plateau is always N = 15 maternal trees and N = 5 maternal trees for upper plateau): (1) site of maternal trees (fixed factor plateau with two levels: ‘upper’ and ‘lower’), (2) distance from maternal tree (fixed factor distance with two levels: ‘close’ and ‘away’), (3) propagule type (fixed factor propagule with two levels: ‘seed’ and ‘fruit’), and (4) protection from vertebrate fruit- or seed predators (fixed factor cage with two levels: ‘cage’ and ‘no cage’). The 20 maternal trees were used as a random factor. This gave a total of 160 groups of seeds or fruits that will be referred to as ‘patches’. Around each of the 20 maternal trees, the four close patches were set up 1 m away from the trunk in the four cardinal compass directions. The four away patches were set up in one of two different ways: either 20–25 m away from the maternal tree in the four cardinal directions, or 20–25 m away in a roughly perpendicular line with at least 6–8 m between patches (Figure S1). None of the away patches were set up closer than 25 m to any other *S. mamillatum* tree. In each of the seed patches we placed 4–7 manually de-pulped seeds. The fruit patches consisted of three whole fruits. Both seeds and whole fruits in any one patch were placed directly on the ground in a 10×10 cm area. The cages were built with 0.5×0.5” wire mesh, 16×16×8 cm in size, and were fixed close to the ground by 6–8 metal pegs around the base (Figure 1H). Cages were removed when the first seedling in a caged patch was about to touch the wire mesh, as we wanted to avoid any physical interference with seedling growth. This was done in October–December 2004, when almost all seedlings had emerged and seed predation was no longer considered important (see Figure 1I for a typical patch of seedlings)

Seeds from the tortoise feeding experiments were also put out in Brise Fér CMA. Whole gut-passed seeds and several large fragments (half a seed, one cotyledon) were put out once a week in two caged plots (‘plot’ hereafter refers only to gut-passed seeds or seedlings), one on the upper plateau and one on the lower plateau. However, since we recovered relatively few whole seeds from the tortoise faeces, we set up only ‘away’ plots. Thus, plots were placed 25–40 m away from any *S. mamillatum* tree, and a minimum of 15 m away from each other. The underlying assumption for this choice being that even a tortoise will likely move a greater distance than 25 m within 1–3 weeks. Each plot consisted of two 15×15 cm sections, one with whole seeds (N = 7–10) and one with seed fragments (N = 10), spread out evenly. The two sections in each plot were roughly 30–40 cm apart and were covered with a ca. 1 cm thick layer of tortoise dung. Each plot was covered with a wire-mesh cage of roughly 100×100×20 cm in size. These cages were removed in December 2004. Two plots were set up each of the first four weeks and four plots were set up in the fifth week, where most gut-passed seeds were collected. Thus, we had a total of 12 plots, with N = 6 on the upper and N = 6 on the lower plateau.

#### Initial seed numbers in patches

As we put out whole fruits in the fruit patches we did not know how many seeds each fruit contained. Thus, we established a baseline number of seeds for each of these patches for use in subsequent analysis of germination patterns and germination success. This was done by scoring the number of whole seeds as soon as the pulp had decomposed, usually after 1–2 months. We investigated effects of propagule, distance, and cage on initial numbers of seeds per patch with an ANOVA.

#### Germination patterns

Seedling germination in patches and plots was recorded six times; once per month for the first four months (where the majority of seeds germinated), and thereafter at different intervals, depending on timing of fieldwork in Mauritius. Germination was defined as the emergence of the first two leaves and not only the root growing into the soil, because many seeds never managed to get past the latter stage and died before extending the shoot.

Due to the different number of maternal trees on the upper and lower plateau, the loss of several patches to feral pigs that broke into the CMA and to weeders working in the CMA, our experimental design was unbalanced. Furthermore, for the calculation and analyses of proportions of seeds germinated we needed to take the number of initial seeds in each patch into account. We therefore analysed seedling germination patterns with a generalised linear mixed-effects model with penalised quasi-likelihood (hereafter GLMM) (glmmPQL function in R.2.4.1, using the MASS library [36]), with plateau, distance, propagule, cage and Time as fixed effects, maternal tree as a random effect, and using a binomial error structure. This method is robust for unbalanced data, and by using the ‘cbind’ command to calculate the germination proportions we weighted the sample sizes (number of seeds and seedlings per patch). Furthermore, we fitted an offset factor to take the different length of time intervals between germination censuses into account. Initially, we fitted the full model, whereupon non-significant higher-order interactions were removed and only the simplified model is presented (using the function ‘anova.lme’ from the nlme library to assess statistical significance).

#### Overall germination success

The overall germination success (proportion of initial seeds that germinated) was analysed by comparing the proportions of maximum number of seedlings out of the initial number of seeds in each patch with a GLMM, using the same fixed (except for Time) and random effects and error structure as above (for almost all patches the maximum number of seedlings was reached around December–January 2004). Initially, we fitted the full model, whereupon non-significant higher-order interactions were removed.

We compared germination success for gut-passed seeds to manually depulped seeds germinating in cages away from maternal trees only (‘away seed cage’ patches), using a GLM with a quasi-binomial error structure to account for over-dispersed data. Again, the underlying assumption for this comparison being that even a tortoise will likely move a greater distance than 25 m within 1–3 weeks.

### Seedling morphometrics

We measured the height (from ground to where the uppermost leaf pair was attached to stem) and counted the leaves of all seedlings in each patch and plot twice (January 2005 and February 2006). For the analysis of the seedlings in patches, we used linear mixed-effects models with patch nested within maternal tree as random factors.

For seedling height in the plots we compared average seedling height per plot with height of seedlings in all patches (there was no significant difference in height between patches, see Results), averaged at the maternal tree level, with a Wilcoxon-Mann-Whitney test. Numbers of leaves per seedling in the plots were compared to numbers of leaves per seedling in away patches only, averaged at the maternal tree level, with a Wilcoxon-Mann-Whitney test. For both analyses, we pooled upper and lower plateau maternal trees and plots, as there were only few plots with seedlings germinating.

### Seedling damage

We here define seedling damage broadly as a visible mark caused by anything that damages and/or feeds on the leaves. Levels of seedling damage were scored twice, in both patches and plots.

In the first survey in early January 2005, we randomly selected one seedling from each of the 160 patches where one or more seedlings had emerged and were still alive at this time (N = 117 patches). Due to the low number of emerged seedlings in the plots with gut-passed seeds, we here scored seedling damage on all seedlings and used plot averages in the analysis. We measured seedling height and counted the number of leaves for each seedling. We assessed the presence or absence of different categories of damage on each leaf, divided into seven categories: 1) leaf mines, 2) necrosis spots, 3) bite damage, 4) discolouration/wilting, 5) curled leaves, 6) fungus, and 7) scale insects. We analysed the proportion of total number of leaves affected by each of the damage categories, as well as the overall proportion of total number of leaves affected by one or more damage categories. We also investigated the diversity of damage categories suffered at the seedling level by analysing the proportion of all seven damage categories present at the seedling level. To weigh these proportions in relation to total number of leaves per seedlings, we used GLMMs with distance and plateau as fixed effects and maternal tree as random effect, and with binomial error structures. A separate model was fitted for each of the seven damage categories, as well as one for overall proportion of damaged leaves and one for diversity of damage at the seedling level. We investigated possible interdependencies between damage categories with Pearson's correlation tests.

Seedling damage in the plots with seedlings from gut-passed seeds was compared to that of away seedling patches, using GLMMs, combining maternal tree and plot into one random effect. Here, we also fitted a separate model for each of the seven damage categories, one for overall proportion of damaged leaves, and one for diversity of damage at the seedling level.

In the second seedling damage survey in mid-February 2005, we visually assessed the overall level of damage for all seedlings in each patch and each plot (N = 117 patches and 7 plots), using the following grouping: 1 = low (almost no damage, most to all seedlings healthy, only few leaves damaged), 2 = medium (little damage, most seedlings healthy with few leaves damaged, one to a few seedlings damaged), and 3 = heavy (damage affecting most seedlings, leaves curled or wilting, one to more seedlings badly affected). For patches, the results of the second survey were analysed with a linear mixed-effects model [37], using distance and plateau as fixed effects and maternal tree as random effect. Seedling damage levels in the plots with seedlings from gut-passed seeds were compared to away patches, averaged at the maternal tree level, with a Student's t-test.

### Seedling survival

Seedling survival was investigated by analysing the proportion of surviving seedlings in February 2006 in relation to the maximum number of seedlings in patches where at least one seedling had germinated (N = 132 patches). We used a GLMM with a binomial error structure. We initially fitted a full model with all factors (fixed: plateau, distance, propagule, cage; random: maternal tree). Any significant or marginally significant (*P*<0.1) factors or interactions between factors were retained, and included in a new minimum adequate model. Again, survival of seedlings in plots was compared to survival of away seedling patches only. We used a GLMM for the analysis, combining maternal tree and plot into one random effect.

## Results

### Feeding experiments with ecological analogue species

**Figure 2:**
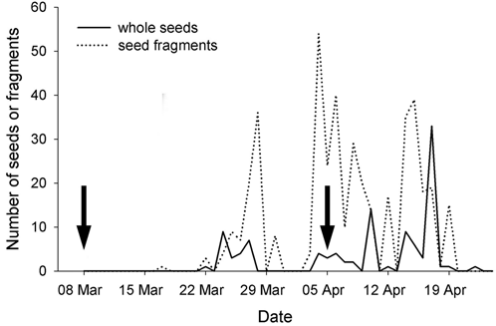
Gut-passage patterns of seeds and seed fragments of *Syzygium mamillatum* fruits fed to Aldabra tortoises. The two arrows indicate the beginning and the end of the feeding period, respectively.

Of the estimated total of 685 seeds fed to the giant tortoises, 108 (15.8%) passed unharmed, and we recovered an additional 419 fragments with a total weight of 143.9 g, corresponding to approximately 197 seeds (28.8%). Thus, an estimated 380 seeds (55.4%) were digested, at least partly. Minimum gut passage time was 12 days (from first feeding March 10 to first seed defecated March 22), with a theoretical maximum of 43 days (from first feeding to last seed defecated April 22). As we fed the giant tortoises continuously over several weeks, we could not calculate a mean gut passage time. However, the temporal distribution patterns of gut-passed seeds and seed fragments in relation to the period of feeding suggests a mean gut passage time of 2–3 weeks (Figure 2).

### Germination experiments

#### Initial seed numbers in patches

Each patch contained 5–6 seeds when it was set up (mean±SD = 5.4±1.6 seeds). There was no significant effect of propagule (*F*_1, 147_ = 0.142, *P* = 0.71) or distance (*F*_1, 147_ = 0.141, *P* = 0.71) on initial numbers of seeds per patch. However, patches with cages held on average more seeds than uncaged patches (5.7±1.6 vs. 5.1±1.5 seeds; *F*_1, 147_ = 4.68, *P* = 0.03). This difference, though, was only found for fruit (cage: 6.0±2.1 seeds, no cage: 4.8±2.0 seeds) and not for seed (cage: 5.3±0.89 seeds, no cage: 5.4±0.79 seeds; cage×propagule: *F*_1, 147_ = 14.23, *P* = 0.02). This suggests that pre-germination predation in patches was mostly restricted to whole fruits.

#### Germination patterns

**Figure 3:**
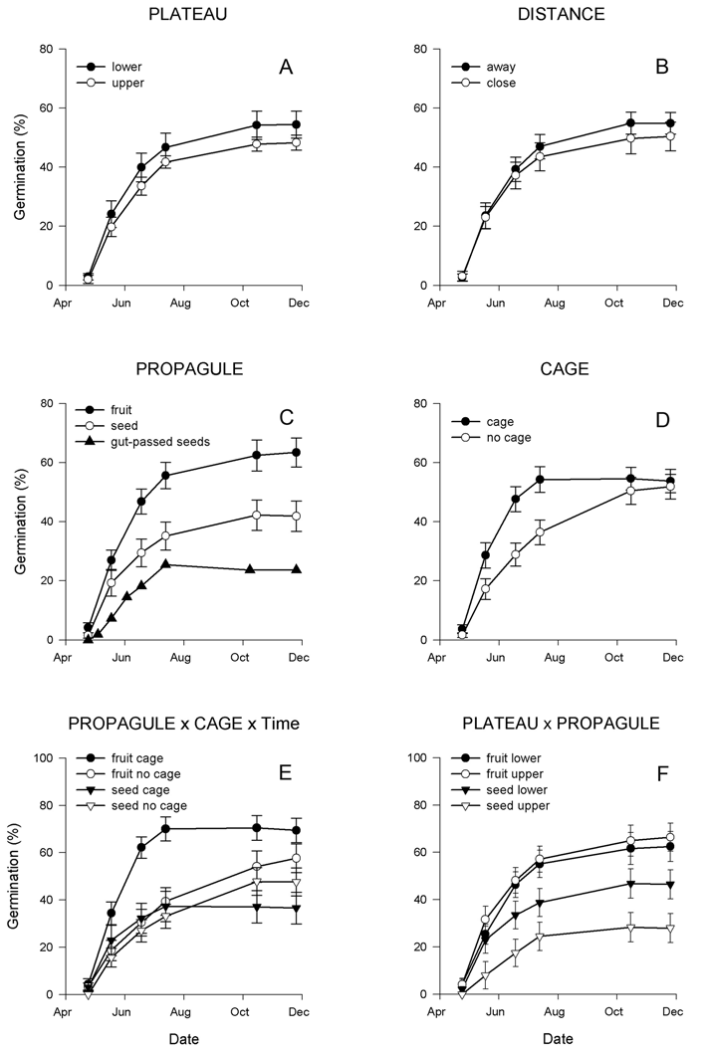
Seed germination patterns of *Syzygium mamillatum*. (A)–(D) illustrates the interactions with Time for each of the main effects (see Table 1). Values plotted are means±1 SE at the maternal tree level (N = 20 trees, except for plateau which a sample size of 15 maternal trees on the lower plateau, and 5 maternal trees on the upper plateau). In (C), we have added the germination pattern of tortoise gut-passed seeds (overall proportion, seeds and seedlings pooled from all 12 gut-passed seed plots). (E) Illustrates the significant interaction from Table 1.

There were no significant main effects of plateau or distance on the overall germination pattern (Figure 3A,B; Table 1). However, seeds from whole fruits germinated both faster and with a higher proportion than manually depulped seeds (Figure 3C; Table 1). Germination was faster with cage than without cage (Figure 3D; Table 1), but only for seeds from whole fruits (Figure 3E; Table 1). Furthermore, there was a significant interaction between propagule and plateau: while there was no difference in germination pattern for seeds from whole fruits on the upper and lower plateau, manually depulped seeds germinated worse on the upper than on the lower plateau (Figure 3F; Table 1).

**Table 1:** *Syzygium mamillatum* seed germination patterns.

	d.f.	*F*	*P*
plateau	1, 18	0.72	0.408
distance	1, 130	0.24	0.627
propagule	1, 130	13.82	**<0.001**
cage	1, 130	1.90	0.171
Time	1, 772	335.98	**<0.001**
plateau×propagule	1, 130	5.75	**0.018**
plateau×distance	1, 130	0.01	0.920
distance×propagule	1, 130	0.03	0.853
plateau×cage	1, 130	1.54	0.218
distance×cage	1, 130	0.12	0.732
propagule×cage	1, 130	5.24	**0.024**
plateau×Time	1, 772	0.03	0.874
distance×Time	1, 772	0.62	0.432
propagule×Time	1, 772	10.89	**0.001**
cage×Time	1, 772	4.64	**0.032**
propagule×cage×Time	1, 772	3.94	**0.048**

Summary of the GLMM used to analyse *S. mammilatum* germination patterns over time; see also Figure 3.

d.f. = numerator degrees of freedom, denominator degrees of freedom; statistical significance indicated with bold.

For gut-passed seeds, there were too few plots (N = 7 plots) where seeds germinated to perform germination pattern analyses with plot as a random factor. However, when plotting the cumulative germination for all gut-passed seeds pooled (Figure 3C, N = 108 seeds), they appeared to germinate more slowly and at a lower proportion than both manually depulped seeds and seeds from whole fruits.

#### Overall germination success

At maternal tree level a grand mean of 60.4±0.03% (all means±1 SE) of the seeds germinated. In the GLMM, the only significant factor was propagule, with mean germination rates being 70.9±0.04% for seeds from whole fruits and 49.3±0.05% for manually depulped seeds (*F*_1, 131_ = 20.86, *P*<0.001). There was a marginally significant interaction between propagule and plateau (*F*_1, 131_ = 2.96, *P* = 0.088), explained by a difference in germination on upper versus lower plateau for manually depulped seeds (upper: 33.4±0.1%, lower: 54.7±0.1%) but not for seeds from whole fruits (upper: 71.4±0.1%, lower: 70.7±0.1%).

**Figure 4:**
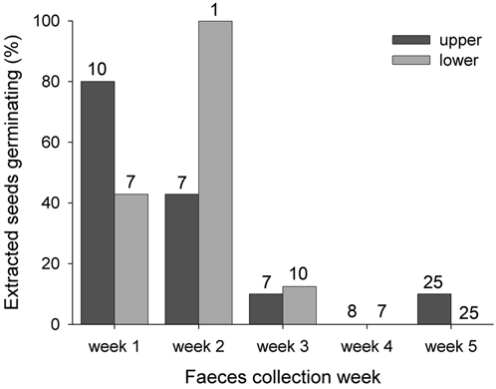
Percentage of tortoise gut-passed seeds germinating in relation to faeces collection week. Numbers above the bars are the number of seeds sown in the forest on the upper and lower plateau, respectively.

Seeds only germinated in seven of the 12 plots, and germination success of the gut-passed seeds in the plots was significantly lower than the ‘away seed cage’ patches used as the control (gut-passed seeds: 18.2±7.0%, control: 47.4±7.6%, *F*_1, 29_ = 6.24, *P* = 0.018). There appeared to be a negative effect of mean gut-passage time on germination success, with the first seeds collected germinating better than the last seeds (Figure 4).

### Seedling morphometrics

In 2005, distance had a highly significant effect on number of leaves per seedling, with more leaves per seedling away (7.6±0.3 leaves) than close (6.2±0.2; *F*_1, 97_ = 12.54, *P*<0.001). There was no effect of distance on seedling height (overall mean height: 69.9±1.4 mm; *F*_1, 97_ = 0.78, *P* = 0.38). The pattern was the same in 2006, with distance affecting number of leaves per seedling (away: 9.2±0.5 leaves; close: 7.5±0.4 leaves; *F*_1, 78_ = 9.15, *P* = 0.003), but not seedling height (overall mean height: 97.0±2.6 mm; *F*_1, 78_ = 0.11, *P* = 0.74). Neither plateau nor plateau×distance interactions were statistically significant for height and number of leaves in 2005 or 2006 (all *P*>0.1).

For seedlings from gut-passed seeds in the plots, we used maternal tree level averages of all patches as control group for height, and away patches as control group for number of leaves (see paragraph above for control values). In 2005, seedlings in plots were of the same height as control seedlings (height in plots: 74.8±4.6 mm, *W* = 42, *P* = 0.30; all tests: N = 6 plots and 20 maternal trees), and had the same number of leaves per seedling as control seedlings (number of leaves in plots: 7.8±0.7 leaves, *W* = 56, *P* = 0.84). When recorded again in 2006, however, seedlings in the plots were significantly larger than control seedlings (height in plots: 124.3±13.4 mm, *W* = 11, *P* = 0.002), and still had significantly more leaves (leaves in plots: 13.4±0.8 leaves, *W* = 14, *P* = 0.006).

### Seedling damage

**Figure 5:**
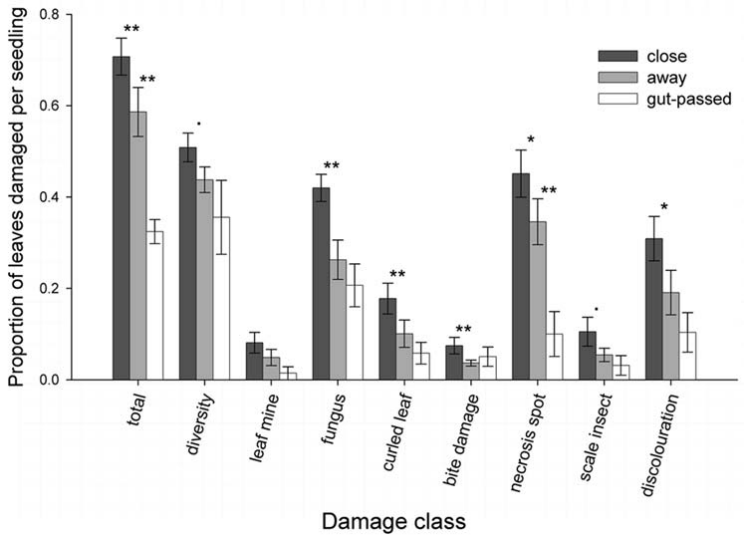
Seedling damage levels recorded in the first seedling damage survey in January 2005. The damage is expressed as proportions of total number of leaves per seedling suffering from the seven individual damage classes: ‘leaf mine’, ‘fungus’, ‘curled leaf’, ‘bite damage’, necrosis’, ‘scale insect’, and ‘discolouration’; ‘total’ means all seven damage classes pooled at the seedling level; ‘diversity’ means how many types of damage a seedling suffers from in proportion out of seven. Significant differences between close and away, and between away and gut-passed are marked above the corresponding pairs of bars (*P*<0.10; * *P*<0.05; ** *P*<0.005; see Table 2). All values for close and away seedlings are means±1 SE at the maternal tree level (N = 20 trees), values for seedlings from gut-passed seeds are means of the seven plots where seedlings emerged.

In the first survey, when we scored the damage at the leaf level for one random seedling per patch, the effect of distance was significant for overall damage level, diversity of damage, and for most of the individual damage categories. A much higher proportion of leaves was damaged close to the maternal trees, compared with seedlings further away (Figure 5; Table 2). Apart from a marginally significant interaction with distance for the damage category scale insects, plateau was not a significant main effect and did not interact with distance for any other damage category.

**Table 2:** First seedling damage survey.

		total		diversity		leaf mine		fungus		curled leaf		bite damage		necrosis spot		scale insect		discolouration	
	d.f.	*F*	*P*	*F*	*P*	*F*	*P*	*F*	*P*	*F*	*P*	*F*	*P*	*F*	*P*	*F*	*P*	*F*	*P*
plateau	1,18	0.46	0.506	0.01	0.941	0.2	0.662	0.11	0.742	0.03	0.876	1.04	0.32	0.08	0.781	0.55	0.468	0.24	0.628
distance	1,93	11.29	**0.001**	3.72	0.057	0.44	0.511	10.94	**0.001**	9.7	**0.002**	7.36	**0.008**	6.01	**0.016**	2.85	0.094	5.73	**0.019**
plateau×distance	1,93	0.79	0.377	0.67	0.415	1.6	0.208	1.62	0.207	0.12	0.725	2.57	0.113	0.88	0.35	2.96	0.089	0.14	0.713
Gut-passed vs. Away	1,25	7.36	**0.012**	0.96	0.337	2.76	0.109	0.7	0.41	0.11	0.749	0.2	0.659	9.01	**0.006**	0.01	0.944	1.26	0.272

The three first lines summarise the nine GLMMs used to analyse the first survey of damage for propagule seedlings, while the lowest line summarises the nine GLMMs used to compare damage levels of seedlings from gut-passed seeds in plots to seedlings in away patches.

d.f. = numerator degrees of freedom, denominator degrees of freedom; statistical significance indicated with bold.

The occurrence of several damage categories were correlated (N = 117 seedlings; *P*-values given after sequential Bonferroni corrections). Presence of white leaf fungus was correlated with presence of leaf mines (*r* = 0.274; *P* = 0.048), curled leaf (*r* = 0.280; *P*<0.036) and necrosis spots (*r* = 0.423; *P*<0.001). Presence of necrosis spots was correlated with presence of scale insects (*r* = 0.335; *P*<0.001), suggesting that the former may be caused by the latter. The least well-defined damage category, discolouration, was correlated with the two damage categories affecting whole leaves, white fungus (*r* = 0.331; *P*<0.001) and curled leaf (*r* = 0.287; *P* = 0.034), and is probably the final stage in overall damage before a leaf wilts and drops off.

Compared with seedlings away from maternal trees, seedlings from gut-passed seeds had a significantly lower total proportion of damaged leaves, whereas there was no difference in the diversity of damage categories (Figure 5, Table 2). While there were trends for seedlings from gut-passed seeds to have a lower proportion of leaves damaged for almost all damage categories, the only significant difference was for necrosis spots (Figure 5, Table 2).

In the second survey, as in the first survey, we found a strong effect of distance, with seedlings in patches close (N = 53) to the maternal trees scoring higher overall levels of damage than seedlings in patches away (N = 64) from the maternal trees (means±1 SE; close: 2.14±0.10; away: 1.62±0.12; linear mixed-effects model: *F*_1,95_ = 22.3, *P*<0.001). Plateau had no significant effect on overall seedling damage level (*F*_1,18_ = 1.92, *P* = 0.18), nor was there a significant interaction between distance and plateau (*F*_1,95_ = 0.003, *P* = 0.95). There was no difference in seedling damage between the seven plots with seedlings from tortoise gut-passed seeds and the away seedling patches (N = 64) used as control group (gut-passed seeds: 1.57±0.20; *F*_1,25_ = 0.008, *P* = 0.93).

### Seedling survival

Seedling survival from when maximum number of seedlings had germinated in a patch (ca. November–December 2004) to February 2006 was strongly influenced by distance and marginally by plateau (Table 3). Distance had a highly significant overall effect on seedling survival, with much fewer seedlings surviving close to maternal trees compared to seedlings further away. Overall, plateau had a marginal effect on seedling survival, with a slightly higher seedling survival on the lower plateau. However, there was a significant interaction between plateau and distance, with seedling mortality being much higher close to maternal trees on the upper plateau than on the lower plateau. Seedlings in the seven plots had the same survival rate as seedlings in the away patches used as a control group (plots: 77.4±13.9%; *F*_1,25_ = 0.020, *P* = 0.89).

**Table 3:** Seedling survival from 2004 to 2006.

Effects	Levels	Survival (%)	d.f.	*F*	*P*
plateau	upper	54.9±7.1	1,18	3.54	0.076
	lower	66.8±4.0			
distance	away	78.1±3.8	1,110	29.49	<0.001
	close	48.0±6.6			
plateau×distance	upper, close	24.5±11.9	1,110	10.37	0.002
	lower, close	55.7±6.2			
	upper, away	88.0±3.7			
	lower, away	75.2±4.6			

Survival calculated from when maximum number of seedlings had germinated in a patch in November–December 2004 to February 2006, with corresponding test statistics from the GLMM analysis. Percentage survival is expressed as means±1 SE at level of the 20 maternal trees, with 15 trees on the lower and 5 trees on the upper plateau.

d.f. = numerator degrees of freedom, denominator degrees of freedom.

Seedling damage recorded at the patch level (second damage survey) in February 2005 was a strong predictor for subsequent mortality from February 2005 to February 2006. Seedlings in patches with low damage level survived significantly better than those in patches with medium and high damage levels (mean survival±1 SE; low: 90.1±3.6%, N = 40 patches; medium: 79.2±4.6%, N = 49 patches; high: 72.8±8.9%; N = 22 patches; GLMM with maternal tree as random factor: *F*_1,91_ = 7.72, *P* = 0.007).

There was no significant effect of number of seedlings in a patch on seedling survival in that patch from maximum number of seedlings in 2004 to February 2006 (GLMM with maternal tree as random factor, and using the maximum seedling number observed per patch: *F*_1,111_ = 0.363, *P* = 0.55).

## Discussion

We found strong negative effects of proximity to maternal trees for seedling growth and survival in the critically endangered endemic Mauritian tree *Syzygium mamillatum*.

This, to our knowledge, provides the first experimental evidence for a Janzen-Connell distance-dependent effect on the growth and survival of seedlings on an oceanic island. Our results clearly demonstrate the crucial importance of *S. mamillatum* propagules being dispersed away from the maternal trees. The fruitless search for natural seedlings and saplings away from adult trees suggests that there are currently no frugivorous animals acting as efficient seed dispersers of *S. mamillatum*. Furthermore, the fact we found no large seedlings or even saplings beneath or in the vicinity of adult trees strongly suggests that beneath maternal trees, survival of seedlings above a certain age (likely some 3–4 years) is very close to zero indeed. Thus, dispersal away from the maternal tree – or probably any adult conspecific – is apparently crucial for this species. We demonstrate that Aldabra giant tortoises could be used as ecological analogues to provide seed dispersal services, and thus resurrect the functional component of some of the extinct endemic frugivores in Mauritius.

### Seed germination, and seedling growth, damage and survival

There was no difference in germination rate or germination speed between close and away patches. However, germination rate and speed, and overall germination success were strongly affected by propagule type. For several Mauritian fleshy-fruited tree species, it has been shown that removal of the fruit pulp is important for successful seed germination; if left on ripe fruits, the pulp often gets infected by fungi that spread into the seeds and destroy them [28], [38]. Seed destruction or reduced seed germination rate as a result of fungal infections of the pulp is a common pattern found elsewhere as well [e.g. 39]. However, this was not the case for *S. mamillatum*. On the contrary, our results show that seeds from whole fruits germinate faster and at a higher rate than manually depulped seeds. This could be due to a high level of essential oils with anti-fungal properties found in many plants from the family of Myrtaceae (e.g. in fruits of *S. cordatum* [40]). Moreover, some *Syzygium* species in Australia germinate better if fruits have been fermenting [41], and a similar effect is possible for *S. mamillatum*. Lastly, because *S. mamillatum* seeds have no hard endocarp, the pulp may protect seeds from rapid desiccation that could force them into dormancy. The latter is likely to be the main explanation in our case, as manually depulped seeds germinated more slowly and at a lower rate on the upper plateau, which is a drier and warmer habitat than the lower plateau. Seeds from whole fruits, on the other hand, germinated equally well on the upper and lower plateau.

The effects of caging on germination were more complex. Initially, the cages did protect the propagules against predation by larger animals. However, this effect was only seen for whole fruits, where initial counts of seeds (after the pulp had rotted away) were lower for non-caged than for caged patches. This is probably due to introduced ground-foraging animals, such as rats or tenrecs, grabbing whole fruits in each non-caged fruit patch. Caging also had a strong effect on germination speed, but only for seeds from whole fruits. This is puzzling, but may be due to foraging animals selectively disturbing decomposing fruit (where seeds often already had started germinating, pers. obs.), and not single seeds.

Most importantly, contrary to seedling germination patterns, seedling damage levels and subsequent seedling survival were strongly affected by proximity to maternal trees. Seedlings suffered less damage and had higher survival rates when growing away from maternal trees. Furthermore, seedlings had more leaves away from maternal trees. The overall poorer growth conditions on the upper plateau, less soil of a worse quality and a drier habitat, were also evident in seedling growth and seedling survival.

### The effects of tortoise gut-passage

Despite the relatively low number of *S. mamillatum* seeds passing undamaged through the tortoises, and despite the lower overall germination success of gut-passed seeds, there are at least two mitigating factors that could contribute to a high overall success for seedlings from gut-passed seeds in the medium to long term: Firstly, seeds are almost certainly dispersed away from areas with high seedling mortality near adult trees. Secondly, gut-passed seeds are deposited in a favourable microclimate with plenty of nutrients, which our results showed leads to better growth and a lower susceptibility to natural enemies. Seedlings from gut-passed seeds grew taller, had more leaves, and suffered less leaf damage than control seedlings in both damage surveys. This could be because the higher nutrient status means that more secondary compounds to deter natural enemies can be produced [42].

Our estimated mean gut-passage time of 2–3 weeks is comparable to results from other studies of giant tortoises [43], [44]. As illustrated in Figure 4, longer gut-passage time appears to decrease germination success. All else being equal, the seeds extracted from tortoise faeces in week five were likely to have spent longer in tortoise guts than seeds extracted in week one. A similar trend towards lower germination success with increasing tortoise gut-passage time was noted for *Lycopersicon cheesmanii* seeds ingested by Galápagos tortoises [43].

Compared to seeds of many other Mauritian fleshy-fruited plant species, seeds of *S. mamillatum* are very unprotected; they have no hard endocarp to protect the cotyledons and embryo, and therefore break apart easily, destroying the seed. In contrast, the seeds of most other Mauritian fleshy-fruited plant species have some sort of harder seed coat that would provide ample protection during tortoise gut-passage. For example, on the small off-shore islet Ile aux Aigrettes, free-ranging Aldabra tortoises eat the fallen fruits of the endangered *Diospyros egrettarum* (Ebenaceae). The seeds of this species have a thin but hard and smooth seed coat and pass through the tortoises unscathed (Figure S2), germinating very well afterwards (pers. obs.). To summarise, our results show how even very fragile seeds can survive tortoise gut-passage, and that subsequent performance of seedlings from gut-passed seeds are better than for seeds that have not been ingested. It is therefore very likely that giant Aldabra tortoises will be able to perform well as seed dispersers of many Mauritian plant species.

Lastly, studies that aim to investigate seed germination and seedling establishment and the influence of gut-passage on endangered species in conservation areas, should do so in the field, rather than in nurseries or greenhouses, where conditions can be very different from those in the field. Rodriguez-Perez *et al.* [45] found that germination rates of a species after gut-passage through birds and lizards could vary greatly between field- and garden sites, sometimes with completely opposite patterns. Furthermore, it is important to include all possible control groups to seeds from gut-passage experiments; that is, not only manually depulped seeds but also whole fruits; a setup that is regrettably still not the norm in most experimental seed dispersal studies [46]. Failure to use a proper protocol may lead to wrong recommendations for future conservation management strategies.

### Janzen-Connell patterns on Mauritius and other oceanic islands

Our results clearly demonstrate that the predictions of the Janzen-Connell model apply to seedling survival of *S. mamillatum* in Mauritius. However, more studies on other plant species in Mauritius and, above all, more studies on other oceanic islands are needed before any generalisations can be made. With our study, we were able to identify some of the potential drivers of Janzen-Connell patterns in *S. mamillatum* seedling damage and subsequent mortality. Parts of the damage were related to activity by insects (mines, scale insects and probably most of the small necrosis spots) and fungi (white fungus and maybe curled leaves). Interdependence of damage categories is very likely, and we found significant positive correlations between occurrences of several seedling damage categories. For example, the presence of leaf fungus was significantly positively correlated with presence of two of the mechanical damage categories, leaf mines and necrosis spots. This is in line with García-Guzman and Dirzo [47], who showed that fungal pathogens in a tropical rainforest required insect-damaged leaves to successfully infect plants.

We cannot explicitly conclude that seedling damage and mortality is primarily related to natural enemies, rather than, for example, seedling competition. However, the patterns of increased damage were evident even in the patches with one solitary seedling or few seedlings that did not grow in a tight clump. Here, seedling densities are likely to be below levels that could lead to seedling competition [4]. In fact, it has been suggested that competition for resources between seedlings is unlikely to be a major contributor to seedling mortality in tropical forests, at least for young seedlings [5].

We were not able to identify the specific natural enemies that caused the seedling damage. This is a limitation of our study, and more investigations on the identity and specificity of natural enemies of plants on islands are much needed [7]. Therefore, we cannot speculate on the overall relative importance of generalists and specialists in our study system.

In general, islands are said to harbour simple ecosystems – in which case we would expect more generalist than specialist natural enemies. However, even generalist natural enemies can also be density- or even distance-responsive [2], [4]. Moreover, with increasing age, islands harbour more species-rich and complex plant communities. In turn, this creates more niches for specialised herbivores. The incidence of specialist herbivores on an oceanic island is therefore likely to depend on the age of the island [48], [49]. As a result, we may expect to find specialist-driven Janzen-Connell patterns more often on old than on young oceanic islands. More studies on the prevalence of generalist and specialist natural enemies on oceanic islands, and how they affect regeneration of plant species, are clearly needed.

### The use of ecological analogue species to resurrect lost seed dispersal interactions on oceanic islands

We assessed the use of Aldadra tortoises by using captive animals for feeding experiments, and subsequently putting seeds and faeces out into the CMA. This is a good approach for initial assessments of the suitability and functioning of ecological analogue seed dispersers. However, it contributes only little to restoring natural dynamics in the forest; ultimately, we need to release candidate ecological analogue species into the habitat in which we want to resurrect the lost interactions.

On Curieuse Island in the Seychelles, translocated Aldabra tortoises readily ate fruits of native plants they had not encountered before [50]. They dispersed seeds of invasive species, too, but this would not pose a problem with tortoises being confined within the fenced and weeded CMAs in Mauritius. One major advantage of using giant tortoises as ecological analogues is that it is relatively easy to monitor them and, if necessary, to add or remove tortoises, thus adjusting their impact on the habitat [23].

There are several important points to consider when selecting candidate species for release as ecological analogues within conservation management areas on oceanic islands. Firstly, although it may be tempting to look for the closest living relative of the extinct species [25], an evolutionarily close extant species is not necessarily a good ecological analogue [23]. That is, close taxonomical affinity does not automatically translate into ecological similarity. In particular, this is the case on oceanic islands that are famous for the large number of adaptive radiations. Secondly, it would not make sense to release ecological analogue species without having addressed the factors that resulted in the extinction of the original species in the first place. The latter point is already the main focus of many CMAs on oceanic islands; introduced predators and invasive competitors have been eradicated or are being controlled or excluded, especially on smaller offshore islets and fenced habitats on main islands [51].

It is ironic that one of the first and best known but poorly executed studies of a plant and its extinct seed disperser – and the use of an ecological analogue species to replace it – is from Mauritius. The famous Dodo and Tambalacoque story [52] has been cited frequently in the ecological literature as an example of a disrupted mutualism, but suffers from serious flaws [53], [27], and fails to demonstrate the ‘obligatory mutualism’ it suggests. Most importantly, there is more than one candidate ghost in the Mauritian frugivore fauna that could have dispersed the Tambalacoque seeds; giant tortoises or giant skinks, for example [53], [54]. There are even extant fruitbats that are capable of dispersing the large fruits (V. Florens, pers. comm.). This story does serve to prove a very important point, though: The majority of seed dispersal mutualisms are not specialised. Only rarely does one plant species depend on one animal species for dispersal, and only rarely does one frugivore depend on one plant species for food [55], [56]. Hence, introducing one ecological analogue seed-dispersing species is likely to benefit more than one plant species.

### Conclusions

Many studies have pointed out the important roles of either disrupted seed dispersal mutualisms [e.g. 57], [58] or natural enemies [59], [60] in the conservation of rare plants. With our study we highlight the combined potentially greater importance of both factors for endangered plants on oceanic islands compared to mainland habitats. Conservation management of endangered plants on oceanic islands should take both missing seed dispersers and the potential for Janzen-Connell patterns in seedling growth and mortality into account. We suggest that one way of mitigating a lack of dispersal and improving seedling performance is to use ecological analogue frugivorous species *in situ*. Furthermore, it is important for future studies to expand on the importance of Janzen-Connell patterns in conservation management areas in Mauritius and other oceanic islands to include other, more common species, to be able to investigate density- as well as distance-dependent effects [61]. Our suggestions for using ecological analogue species in the conservation management of endangered oceanic island species may be expanded to mainland habitat fragments, which often suffer from locally extinct seed dispersal interactions [e.g. 62], [63]. A broad empirical and experimental assessment of the use of ecological analogues in such habitats in the near future may pave the way for a broader acceptance of the use of ecological analogues in larger tracts of mainland habitat. Here, current seed dispersal dynamics suffer not only from the Pleistocene loss of megafauna [16], [64], [65], but also from ongoing defaunation of other animals (as highlighted in a recent special section of the journal Biotropica [66]). Lastly, our work highlights how the study of critically endangered species can advance both basic research and applied conservation biology.

## Supporting Information

Figure S1 Experimental design of seed germination experiment, with ‘patches’ around the 20 focal maternal *Syzygium mamillatum* trees (not to scale). ‘S’ and ‘F’ denotes patches with seeds and whole fruits, respectively. Shading represents cages, that were used during the first few months of germination and seedling growth. ‘Away’ patches were set up 20–25 m away from maternal tree in one of two ways, depending on distance to nearest adult *S. mamillatum*: ‘Away A’ - in the four cardinal directions, or ‘Away B’ - in a perpendicular line with 6–8 m between patches.
(0.07 MB TIF)

Figure S2 Aldabran giant tortoise dispersing ebony seeds. (A) In the nature reserve on the offshore Mauritian island Ile aux Aigrettes, released free-roaming giant Aldabran tortoises *Aldabrachelys gigantea* eat fruits of the endangered endemic ebony *Diospyros egrettarum*. (B, C) In the fruiting season, one tortoise turd can contain up to several hundred seeds, the vast majority of which have survived the gut passage unscathed. Formerly restricted to one small patch on the 25-ha island, young ebony seedlings can now be found widespread across much of the island, attesting to the potential of *A. gigantea* as ecological analogues for the two extinct Mauritian giant tortoises.
(6.94 MB TIF)

## References

[1] Howe HF, Miriti MN (2000). No question: seed dispersal matters.. TREE.

[2] Janzen DH (1970). Herbivores and the number of tree species in tropical forests.. Am Nat.

[3] Connell JH (1971). On the role of natural enemies in preventing competitive exclusion in some marine animals and in rain forest trees, in Boer PJD, Gradwell GR, Editors. Dynamics of populations..

[4] Clark DA, Clark DB (1984). Spacing dynamics of a tropical rain forest tree: Evaluation of the Janzen-Connell model.. Am Nat.

[5] Wright SJ (2002). Plant diversity in tropical forests: a review of mechanisms of species coexistence.. Oecologia.

[6] Novotny V, Basset Y (2005). Host specificity of insect herbivores in tropical forests.. Proc Roy Soc B.

[7] Ribeiro SP, Borges PAV, Gaspar C, Melo C, Serrano ARM (2005). Canopy insect herbivores in the Azorean Laurisilva forests: key host plant species in a highly generalist insect community.. Ecography.

[8] Cox PA, Elmqvist T, Pierson ED, Rainey WE (1991). Flying foxes as strong interactors in South Pacific island ecosystems: a conservation hypothesis.. Cons Biol.

[9] Lawton J (1995). Ecology of the afterlife.. Oikos.

[10] Clark DA, Clark DB (1981). Effects of seed dispersal by animals on the regeneration of *Bursera gravevolens* (Burseraceae) on Santa Fe Island, Galápagos.. Oecologia.

[11] Lee MAB (1985). The dispersal of *Pandanus tectorius* by the land crab *Cardisoma carnifex*.. Oikos.

[12] Wiles GJ, Schreiner IH, Nafus D, Jurgensen LK, Manglona JC (1996). The status, biology, and conservation of *Serianthes nelsonii* (Fabaceae), an endangered Micronesian tree.. Biol Cons.

[13] Arevalo JR, Fernandez-Palacios JM (2003). Spatial patterns of trees and juveniles in a laurel forest of Tenerife, Canary Islands.. Pl Ecol.

[14] Vitousek PM, Loope LL, Adsersen H (1995). Islands: biological diversity and ecosystem function: Ecological Studies, v. 115..

[15] Grant PR (1998). Evolution on islands:.

[16] Galetti M (2004). Parks of the Pleistocene: recreating the Cerrado and the Pantanal with megafauna.. Natureza & Conservação.

[17] Martin PS (2005). Twilight of the mammoths - Ice age extinctions and the rewilding of America, California University Press..

[18] Zimov SA (2005). Pleistocene park: Return of the mammoth's ecosystem.. Science.

[19] Donlan J, Greene HW, Berger J, Bock CE, Bock JH (2005). Re-wilding North America.. Nature.

[20] Donlan CJ, Berger J, Bock CE, Bock JH, Burney DA (2006). Pleistocene rewilding: An optimistic agenda for twenty-first century conservation.. Am Nat.

[21] Rubenstein DR, Rubenstein DI, Sherman PW, Gavin TA (2006). Pleistocene park: Does re-wilding North America represent sound conservation for the 21st century?. Biol Cons.

[22] Caro T (2007). The Pleistocene re-wilding gambit.. TREE.

[23] Jones CG, Perrow MR, Davy AJ (2002). Reptiles and amphibians.. Handbook of Ecological Restoration.

[24] Steadman DW, Martin PS (2003). The late Quaternary extinction and future resurrection of birds on Pacific islands.. Earth-Sci Rev.

[25] Hutton I, Parkes JP, Sinclair ARE (2007). Reassembling island ecosystems: the case of Lord Howe Island.. Anim Cons.

[26] Maunder M, Page W, Mauremootoo J, Payendee R, Mungroo Y (2002). The decline and conservation management of the threatened endemic palms of the Mascarene Islands.. Oryx.

[27] Cheke AS, Hume JP Lost land of the Dodo..

[28] Nyhagen DF, Turnbull SD, Olesen JM, Jones CG (2005). An investigation into the role of the Mauritian flying fox, *Pteropus niger*, in forest regeneration.. Biol Cons.

[29] Bosser J, Cadet T, Guého J (1987). Nouvelles observations sur des *Syzygium* (Myrtaceae) des Mascareignes.. Adansonia.

[30] Kaiser CN, Hansen DM, Müller CB (2008). Habitat structure affects reproductive success of the rare endemic tree *Syzygium mamillatum* (Myrtaceae) in restored and unrestored sites in Mauritius.. Biotropica.

[31] Lorence DH, Sussman RW (1986). Exotic species invasion into Mauritius wet forest remnants.. J Trop Ecol.

[32] R Development Core Team (2006). R: A language and environment for statistical computing..

[33] Cheke AS, Diamond AW (1987). An ecological history of the Mascarene Islands, with particular reference to extinctions and introductions of land vertebrates.. Studies of Mascarene Island birds.

[34] Hachisuka M (1953). The Dodo and kindred birds, or the extinct birds of the Mascarene Islands..

[35] Hnatiuk SH (1978). Plant dispersal by the Aldabran giant tortoise, Geochelone gigantea (Schweigger).. Oecologia.

[36] Venables WN, Ripley BD (2002). Modern applied statistics with S..

[37] Pinheiro JC, Bates DM (2000). Mixed-effects models in S and S-PLUS..

[38] Wyse-Jackson PS, Cronk QCB, Parnell JAN (1988). Notes on the regeneration of two rare Mauritian endemic trees.. Trop Ecol.

[39] Oliveira PS, Galetti M, Pedroni F, Morellato LPC (1995). Seed cleaning by *Mycocepurus goeldii* ants (Attini) facilitates germination in *Hymenaea courbaril* (Caesalpiniaceae).. Biotropica.

[40] Pretorius JC, Zietsman PC, Eksteen D (2002). Fungitoxic properties of selected South African plant species against plant pathogens of economic importance in agriculture.. Ann Appl Biol.

[41] Beardsell DV, O'Brien SP, Williams EG, Knox RB, Calder DM (1993). Reproductive biology of Australian Myrtaceae.. Aust J Bot.

[42] Coley PD, Bryant JP, Chapin FS (1985). Resource availability and plant antiherbivore defense.. Science.

[43] Rick CM, Bowman RI (1961). Galápagos tomatoes and tortoises.. Evolution.

[44] Hamilton J, Coe M (1982). Feeding, digestion and assimilation of a population of giant tortoises (*Geochelone gigantea* (Schweigger)) on Aldabra atoll.. J Arid Env.

[45] Rodríguez-Perez J, Riera N, Traveset A (2005). Effect of seed passage through birds and lizards on emergence rate of mediterranean species: differences between natural and controlled conditions.. Funct Ecol.

[46] Samuels IA, Levey DJ (2005). Effects of gut passage on seed germination: do experiments answer the questions they ask?. Funct Ecol.

[47] García-Guzman G, Dirzo R (2001). Patterns of leaf-pathogen infection in the understory of a Mexican rain forest: incidence, spatiotemporal variation, and mechanisms of infection.. Am J Bot.

[48] Borges PAV, Brown VK (1999). Effect of island geological age on the arthropod species richness of Azorean pastures.. Biol J Linn Soc.

[49] Gillespie RG, Roderick GK (2002). Arthropods on islands: Colonization, speciation, and conservation.. Ann Rev Entomol.

[50] Hambler C (1994). Giant tortoise *Geochelone gigantea* translocation to Curieuse Island (Seychelles): success or failure?. Biol Cons.

[51] Veitch CR, Clout MN (2002). Turning the tide: The eradication of invasive species..

[52] Temple SA (1977). Plant-animal mutualism: coevolution with Dodo leads to near extinction of plant.. Science.

[53] Witmer MC, Cheke AS (1991). The dodo and the tambalacoque tree: an obligate mutualism reconsidered.. Oikos.

[54] Iverson JB (1987). Tortoises, not Dodos, and the Tambalacoque tree.. J Herpetol.

[55] Howe HF, Smallwood J (1982). Ecology of seed dispersal.. Ann Rev Ecol Syst.

[56] Bascompte J, Jordano P, Olesen JM (2006). Assymetric coevolutionary networks facilitate biodiversity maintenance.. Science.

[57] Bond WJ (1994). Do mutualisms matter? Assessing the impact of pollinator and disperser disruption on plant extinction.. Phil Trans Roy Soc Lond B.

[58] Traveset A, Riera N (2005). Disruption of a plant-lizard seed dispersal system and its ecological effects on a threatened endemic plant in the Balearic Islands.. Cons Biol.

[59] Gilbert GS, Hubbell SP (1996). Plant diseases and the conservation of tropical forests.. BioScience.

[60] Bevill RL, Louda SM, Stanforth LM (1999). Protection from natural enemies in managing rare plant species.. Cons Biol.

[61] Wills C, Condit R, Foster RB, Hubbell SP (1997). Strong density- and diversity-related effects help to maintain tree species diversity in a neotropical forest.. PNAS.

[62] Cordeiro NJ, Howe HF (2001). Low recruitment of trees dispersed by animals in African forest fragments.. Cons Biol.

[63] Galetti M, Donatti CI, Pires AS, Guimarães PR, Jordano P (2006). Seed survival and dispersal of an endemic Atlantic forest palm: the combined effects of defaunation and forest fragmentation.. Bot J Linn Soc.

[64] Janzen DH, Martin PS (1982). Neoptropical anachronisms: The fruits the Gomphoteres ate.. Science.

[65] Guimaraẽs PR, Galetti M, Jordano P (2008). Seed dispersal anachronisms: rethinking the fruits extinct megafauna ate.. PLoS ONE.

[66] Wright SJ, Stoner KE, Beckman N, Corlett RT, Dirzo R (2007). The plight of large animals in tropical forests and the consequences for plant regeneration.. Biotropica.

