# Supplementary figures and images for: Seed Dispersal and Establishment of Endangered Plants on Oceanic Islands: The Janzen-Connell Model, and the Use of Ecological Analogues
Source: PLoS One. 2008 May 7;3(5):e2111. doi: 10.1371/journal.pone.0002111 (PMC2358974; doi:10.1371/journal.pone.0002111)

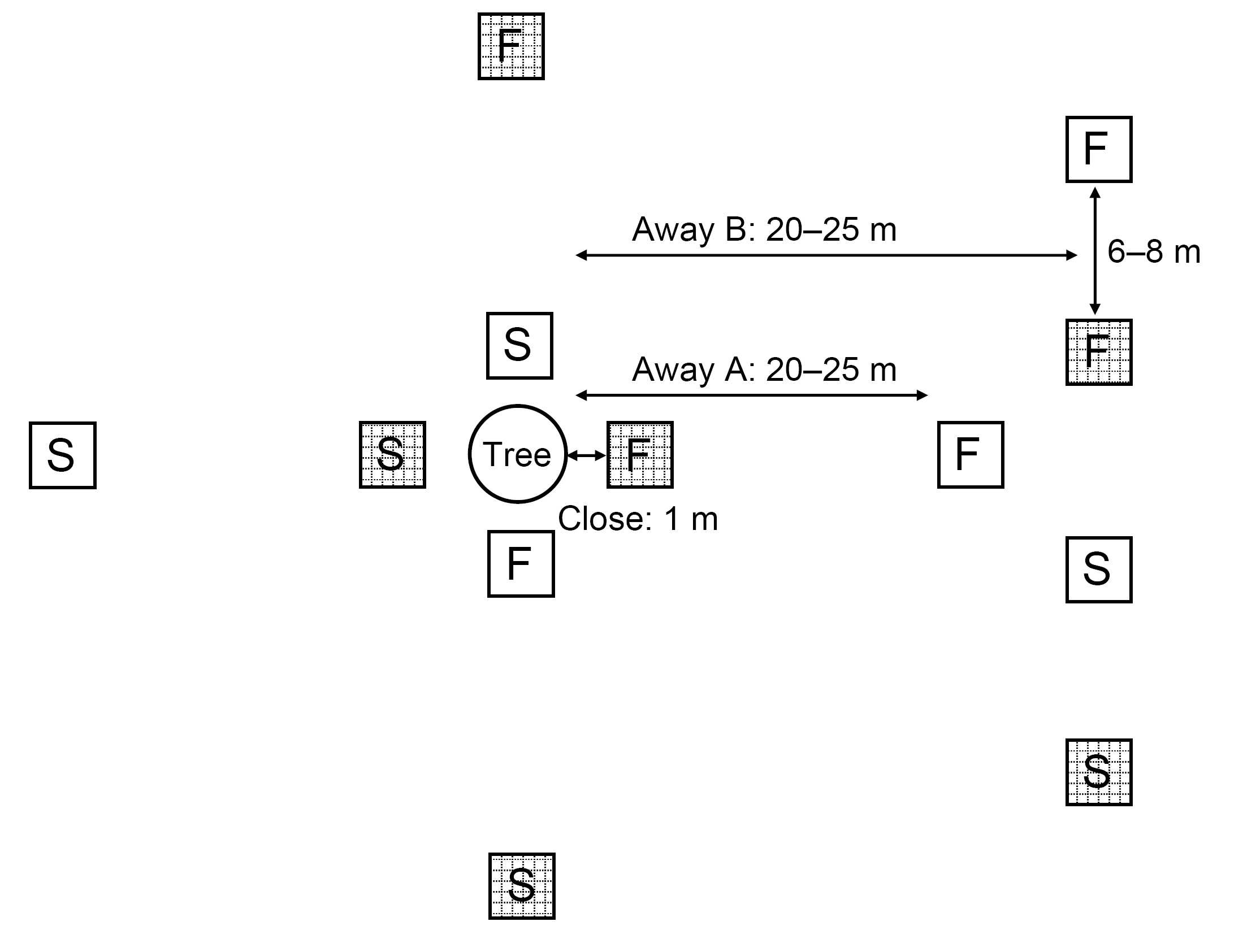

Supplement: Figure S1 — Experimental design of seed germination experiment, with ‘patches’ around the 20 focal maternal Syzygium mamillatum trees (not to scale). ‘S’ and ‘F’ denotes patches with seeds and whole fruits, respectively. Shading represents cages, that were used during the first few months of germination and seedling growth. ‘Away’ patches were set up 20–25 m away from maternal tree in one of two ways, depending on distance to nearest adult S. mamillatum: ‘Away A’ - in the four cardinal directions, or ‘Away B’ - in a perpendicular line with 6–8 m between patches. (0.07 MB TIF) [file pone.0002111.s001.tif]

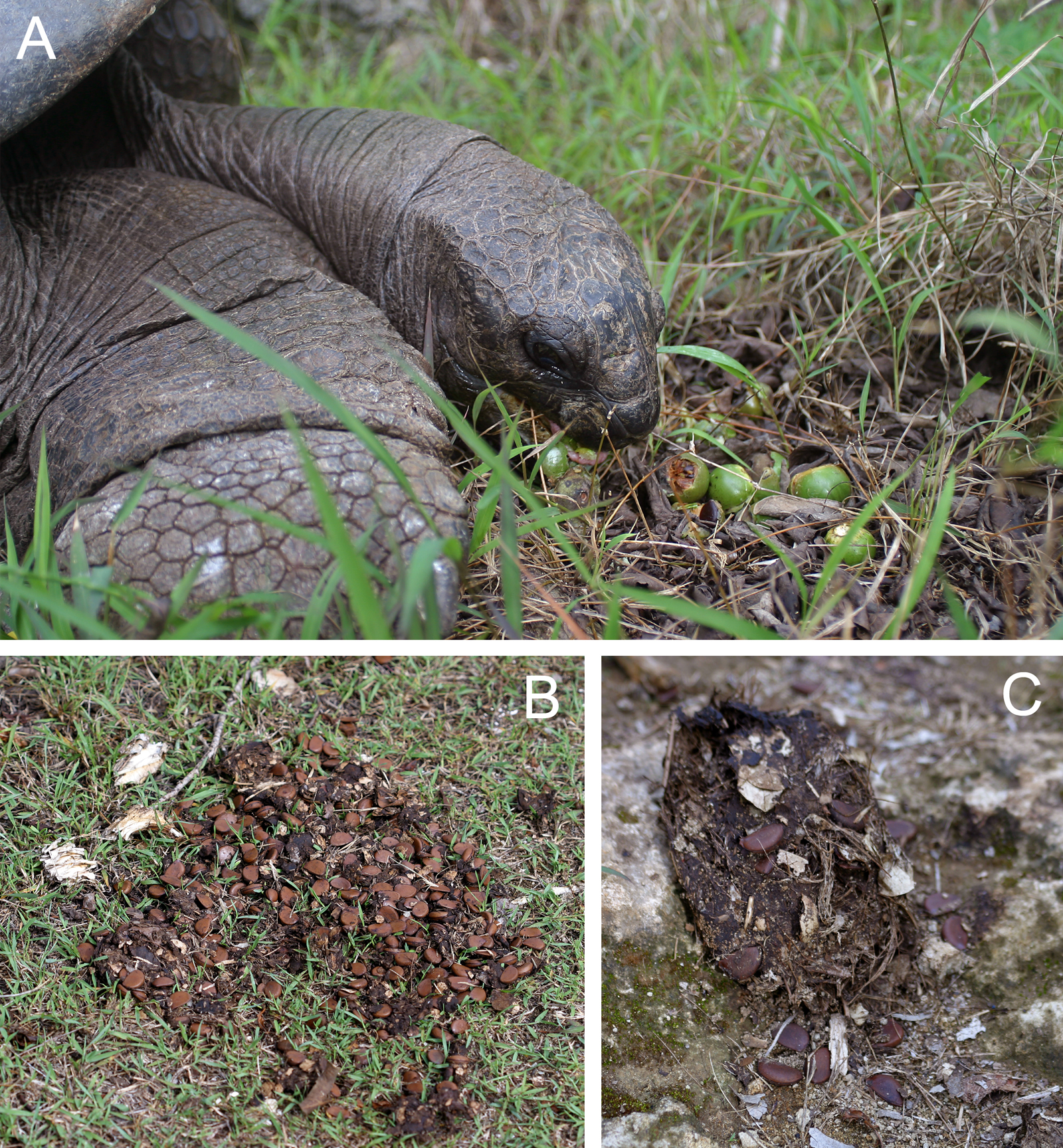

Supplement: Figure S2 — Aldabran giant tortoise dispersing ebony seeds. (A) In the nature reserve on the offshore Mauritian island Ile aux Aigrettes, released free-roaming giant Aldabran tortoises Aldabrachelys gigantea eat fruits of the endangered endemic ebony Diospyros egrettarum. (B, C) In the fruiting season, one tortoise turd can contain up to several hundred seeds, the vast majority of which have survived the gut passage unscathed. Formerly restricted to one small patch on the 25-ha island, young ebony seedlings can now be found widespread across much of the island, attesting to the potential of A. gigantea as ecological analogues for the two extinct Mauritian giant tortoises. (6.94 MB TIF) [file pone.0002111.s002.tif]
